# 1-Pyrroline-5-carboxylate inhibit T cell glycolysis in prostate cancer microenvironment by SHP1/PKM2/LDHB axis

**DOI:** 10.1186/s12964-024-01493-1

**Published:** 2024-02-08

**Authors:** Lei Chang, Guohao Li, Shan Jiang, Jie Li, Jin Yang, Kavita Shah, Le Zhou, Hanrui Song, Leyuan Deng, Zhiguo Luo, Yonglian Guo, Yutao Yan

**Affiliations:** 1 Department of Urology, Central Hospital of Wuhan, Tongji Medical College, Huazhong University of Science and Technology, Wuhan, 430014 China; 2 Department of Clinical Oncology, Taihe Hospital, Hubei University of Medicine, Shiyan, China; 3 Department of Laboratory Medicine, Taihe Hospital, Hubei University of Medicine, Shiyan, China; 4 Institute of Infection and Immunity, Taihe Hospital, Hubei University of Medicine, Shiyan, China; 5 Department of Chemistry and Purdue University Center for Cancer Research, Purdue University, West Lafayette, IN USA; 6 First Clinical College of College of Medicine and Nursing, Hubei University of Medicine, Shiyan, China

**Keywords:** P5C, T cell, Prostate cancer, SHP1, Glycolysis

## Abstract

**Background:**

Our previous studies demonstrated that 1-Pyrroline-5-carboxylate (P5C) released by prostate cancer cells inhibits T cell proliferation and function by increasing SHP1 expression. We designed this study to further explore the influence of P5C on T cell metabolism, and produced an antibody for targeting P5C to restore the functions of T cells.

**Method:**

We co-immunoprecipated SHP1 from T cells and analyzed the proteins that were bound to it using liquid chromatography mass spectrometry (LC/MS-MS). The influence of P5C on T cells metabolism was also detected by LC/MS-MS. Seahorse XF96 analyzer was further used to identify the effect of P5C on T cells glycolysis. We subsequently designed and produced an antibody for targeting P5C by monoclonal technique and verified its effectiveness to restore the function of T cells in vitro and in vivo.

**Result:**

PKM2 and LDHB bind SHP1 in T cells, and P5C could increase the levels of p-PKM2 while having no effect on the levels of PKM2 and LDHB. We further found that P5C influences T cell energy metabolism and carbohydrate metabolism. P5C also inhibits the activity of PKM2 and decreases the content of intracellular lactic acid while increasing the activity of LDH. Using seahorse XF96 analyzer, we confirmed that P5C remarkably inhibits glycolysis in T cells. We produced an antibody for targeting P5C by monoclonal technique and verified that the antibody could oppose the influence of P5C to restore the process of glycolysis and function in T cells. Meanwhile, the antibody also inhibits the growth of prostate tumors in an animal model.

**Conclusion:**

Our study revealed that P5C inhibits the process of glycolysis in T cells by targeting SHP1/PKM2/LDHB complexes. Moreover, it is important that the antibody for targeting P5C could restore the function of T cells and inhibit the growth of prostate tumors.

**Supplementary Information:**

The online version contains supplementary material available at 10.1186/s12964-024-01493-1.

## Introduction

The proliferation and activation of T cells depend on important metabolic pathways, as they require plenty of nutrients such as saccharides and amino acids [1]. It is well accepted that the T cells in the tumor microenvironment are influenced by the metabolism of cancer cells. Accordingly, the rapid growth of cancer cells inevitably generates huge amounts of metabolic waste such as lactic acid and carbamide, which impair T cells [2, 3]. Previously, we demonstrated that P5C, an N-substituted amino acid, released by prostate cancer cells could inhibit the proliferation and function of T cells [4]. The conversion of proline into P5C is catalyzed by Proline dehydrogenase (PRODH) both during proline biosynthesis and catabolism [5, 6]. Our previous study revealed that P5C inhibits the activity of complex III of the electron transport chain in T cell mitochondria, resulting in increased reactive oxygen species (ROS) levels, which in turn decreases the production of ATP [4]. Hence, our study demonstrated that P5C inhibits the energy metabolism of T cells.

The progression through different steps of T-cell development, activation, and effector functions is tightly bound to specific cellular metabolic processes [7]. Previous studies have established that T-effector cells have a metabolic bias toward aerobic glycolysis, whereas naive and regulatory T cells mainly rely on oxidative phosphorylation [7]. T cells increase their proliferation rate and engage in anabolic metabolic pathways such as glycolysis, fatty acid synthesis, and mitochondrial metabolism to support a transcriptional program of differentiation toward a specific effector subset [8]. Aerobic glycolysis is similarly induced in T cells following TCR activation with co-stimulation and is limited to establish the extent of T cell inflammatory function and proliferation [9]. Activated CD4^+^ T cells are characterized by a high degree of glucose uptake and metabolism [7]. Chemical or genetic inhibition of specific steps in the glycolytic pathway leads to a drastic reduction in T effector-cell proliferation and cytokine production [8, 10, 11]. Therefore, we hypothesize that P5C may inhibit glycolysis and energy metabolism to suppress T cell functions.

In our previous study, we also found that P5C increases the expression of SHP1 in T cells [4]. SHP1 phosphatase is a negative regulator of T cell signaling, acting, at least in part, directly or indirectly through the inactivation of SRC-family kinases [12]. SHP1 knock-down promotes T cell proliferation [13]. SHP1 is recruited by inhibitory receptors PD-1 and CTLA-4 on T cell surface. Tai et al. reported that SHP1 inhibits pyruvate kinase M2 (PKM2) by dephosphorylation, thus inhibiting the Warburg effect and cell proliferation caused by PKM2 [14]. Pyruvate kinase (PK) regulates the final step of glycolysis by transferring a phosphate group from phosphoenolpyruvate (PEP) to ADP to produce pyruvate and ATP [15]. In the cytoplasm, PKM2 is normally present as a homotetramer that allows for the highly effective conversion of glucose to lactate, thus acting as a metabolite kinase. In contrast, the low activity homodimer form of PKM2 uses PEP as a phosphate donor to phosphorylate tyrosine residues, thereby acting as a signal transducer and activator of transcription [16, 17]. These findings led us to speculate that P5C perhaps inhibits T cells’ glycolysis by promoting SHP1 expression and inhibiting the activity of PKM2.

In this work, we further explored whether P5C could suppress T cell proliferation and function by inhibiting cellular glycolysis, while decreasing the content of intracellular lactic acid. As expected, we observed that P5C inhibited T cell glycolysis through SHP1 and PKM2 complexes. These results provide a mechanistic rationale to produce an antibody against P5C, which could be used to restore T cell glycolysis and inhibit the growth of prostate tumors.

## Methods

### Ethics

All of the animal experiments were performed in accordance with the guidelines and animal use regulations of the Hubei University of Medicine and were approved by the Institutional Animal Care and Use Committee (IACUC) of the Hubei University of Medicine (Hubei University of Medicine -No.2023-098). This study conforms to the NIH Guide for the Care and Use of Laboratory Animals (NIH publication no. 85 − 23, revised 2011). Reporting of the procedures and results followed the Animal Research: Reporting of In Vivo Experiments (ARRIVE) guidelines.

### Cell preparation and culture

Human primary CD3^+^ T cells were isolated from healthy people’s blood using a CD3^+^ Microbead kit (Miltenyi Biotec Inc. CA, USA), according to the manufacturer’s instructions. The purity of the CD3^+^ T-cell preparation was assessed by flow cytometry (FACS Calibur, Becton, Dickinson and Company, Franklin Lakes, NJ, USA) using PE-anti mouse CD3 monoclonal antibody (mAb) (eBioscience, Inc., San Diego, CA, USA).

Jurkat cells (human T cell line), LNCaP and PC-3 (human prostate cancer cell lines), were cultured in RPMI-1640 (GE Healthcare Life Sciences Hyclone Laboratories, Logan, UT, USA) supplemented with 10% fetal bovine serum (FBS; GE Healthcare Life Sciences Hyclone Laboratories), 10 µg/mL penicillin and 10 µg/mL streptomycin (Beijing Solarbio Science & Technology Co., Ltd. Beijing, China). The cell culture was maintained in an incubator at 37 °C with 5% CO_2_.

### Cell proliferation assay

Primary T-cell proliferation was determined by a carboxyfluorescein succinimidyl ester (CFSE) (Life Technologies, Carlsbad, CA, USA) proliferation assay according to the manufacturer’s instructions. In brief, CD3^+^ T cells were resuspended in CFSE (5 µM) buffer, incubated at 37 ℃ with 5% CO_2_ for 20 min, and washed twice in complete medium. The stained T cells at 2 × 10^5^ cells per well of a 96-well round-bottom plate were then stimulated with anti-human CD3/CD28 beads (2.5 µl/1 × 10^5^ cells) for the indicated times. The cells were then collected and detected with CFSE by flow cytometry (BD FACS Calibur). Proliferation of Jurkat cells was measured using a cell counting kit-8 (CCK-8, Dojindo Laboratories, Japan) according to the manufacturer’s protocol.

### Cytokine analysis

Levels of IL-2, IL-4, IL-6, IL-10, TNF-α, IFN-γ and IL-17 in the supernatant of cultured CD3^+^ T cells were determined using a Cytometric Beads Array (BD Pharmingen) according to the manufacturer’s instructions. Data were acquired on FACS Canto-II (BD Bioscience, USA). Data were analyzed using FCAP Array software (BD Bioscience).

### Western blot analysis

Cell lysates were prepared in RIPA lysis buffer with the addition of a mammalian protease inhibitor cocktail (Sigma) and phosphatase inhibitor. Protein concentration was measured by BCA protein assay kit. Equal amounts of cell lysates were electrophoresed on SDS-polyacrylamide gels and transferred by electroblotting onto a polyvinylidene fluoride (PVDF) membrane. The following primary antibodies were used: mouse SHP1 (1:1000; abs158291, ABsin), and rabbit PKM2 (1:1000; 4053, CST Technology), p-PKM2 (1:1000; 3827, CST Technology), LDHB (1:1000; A5131, ABclonal), LDHA (1:1000; A1146, ABclonal). Membranes were then incubated with horseradish peroxidase-conjugated secondary antibodies (1:10000; ABclonal Technology). After one-hour incubation, membranes were washed three times with TBST, and then visualized by immunoblotting.

### Co-immunoprecipitation (Co-IP)

The SHP1, PKM2 and LDHB interactome was captured from Jurkat cells using rabbit SHP1 (1:50; 24546-1-AP, Proteintech), and mouse PKM2 (1:50; sc-365,684, Santa Cruz Biotechnology) and LDHB antibodies (1:50; sc-100,775, Santa Cruz Biotechnology). Mouse IgG antibodies (abs955, Absin) were used as a control for non-specific binding proteins. Protein A/G agarose beads (10 µl) was washed twice with 200 µl PBS buffer, and incubated with 1 µl antibody prepared in PBS at room temperature for 30 min on a mixer. The lysed cells were centrifuged for 2 min at 12,000 × *g* and the supernatant was collected. The entire supernatant was mixed with 10 µl of beads with antibody and incubated at 4 °C on a rocker platform overnight. The beads were centrifuged for 1 min at 12,000 × *g* and the supernatant was discarded. The beads were washed three times with 1 ml of cold wash buffer. The beads were then resuspended in 60 µl of electrophoresis sample buffer and heated to 95 °C for 5 min. The beads were centrifuged for 1 min at 12,000 × *g* and the supernatant collected and used for MS analysis or western blotting.

### Proteomics and data analysis

Liquid chromatography mass spectroscopy (LC/MS-MS) analyses were performed by reverse phase chromatography on an Ultimate3000 system. The peptide samples were diluted to 1 µg/µL on the machine buffet. We set the sample volume to 5 µL and collected in the scan mode for 60 min. The peptides were scanned with a mass-to-charge ratio of 350–1200 in the samples. The mass spectrometry data were collected using the Triple TOF 5600 + LC/MS system (AB SCIEX, USA). The peptide samples were dissolved in 2% acetonitrile/0.1% formic acid, and analyzed using the Triple TOF 5600 plus mass spectrometer coupled with the Eksigent nanoLC system (AB SCIEX, USA). The peptide solution was added to the C18 capture column (3 μm, 350 μm×0.5 mm, AB Sciex, USA), and the C18 analytical column (3 μm, 75 μm×150) was applied with a 60 min time gradient and a flow rate of 300 nL/min. mm, Welch Materials, Inc) for gradient elution. The two mobile phases are buffer A (2% acetonitrile/0.1% formic acid/98% H_2_O) and buffer B (98% acetonitrile/0.1% formic acid/2% H_2_O). For IDA (Information Dependent Acquisition), the MS spectrum was scanned with an ion accumulation time of 250 ms, and the MS spectrum of 30 precursor ions was acquired with an ion accumulation time of 50 ms. Collect MS1 spectrum in the range of 350–1200 m/z, and collect MS2 spectrum in the range of 100–1500 m/z. The precursor ion dynamic exclusion time was set to 15 s. We submitted the original MS/MS files from the mass spectrometer to ProteinPilot (https://sciex.com.cn/products/software/proteinpilot-software, version 4.5, SCIEX, Redwood City, California, USA) for data analysis. For the identified protein results, we selected certain filtering criteria, and peptides with an unused score > 1.3 (a credibility of more than 95%) were considered credible peptides, and proteins containing at least one unique peptide were retained.

### Metabolomics analysis

Jurkat cells (1 × 10^5^ per well of a 6 well plate) treated with P5C or SHP1 knockdown for 48 h were harvested and stored at -80 °C. Each group had 10 duplicates. Untargeted metabolite profiling was performed using LC/MS-MS as previously described [4]. The clean data was obtained by the molecular feature extraction (MFE) tool in the Agilent Masshunter Qualitative Analysis B.04.00 software (Agilent Technologies, USA), then was analyzed by PCA (Principal Component Analysis), PLS-DA (Partial least square-discriminant analysis) and OPLS-DA (Orthogonal Partial least square discriminant analysis) methods. Differences between experimental groups were evaluated by an unpaired t test (equal or unequal variance) with VIP (Variable Importance in the Projection) > 1 in PLS-DA model. The levels of statistically significant were set at 95% level (*P* < 0.05). The enrichment analysis was performed based on the hypergeometric test. For KEGG (Kyoto Encyclopedia of Genes and Genomes), the hypergeometric distribution test was performed with the unit of pathway; for GO (gene ontology), it was performed based on the GO term.

### Enzymatic activity and lactate measurements

The activities of PKM2 and LDH were measured using PKM2 and LDH assay kits (abs580073, abs580007; Absin), respectively, according to the manufacturer’s instructions. The extracellular and intracellular lactate levels were measured using a Lactate production assay (abs580160; Absin) according to the manufacturer’s instructions.

### Transfection

Human SHP1 shRNA was cloned in pHBAd vector. The primer sequences are included in Supplementary Table 1. Then adenoviruses were packaged and produced, and transfected into 293 A cells to amplify. After purification, Jurkat cells were transfected with adenoviruses.

### Production of P5C antibody

P5C was purchased from Aikang Biotechnolgy (Nanjing, China) and dissolved in HCl solution. The concentration of P5C used in cell experiments was 10 nM. We prepared 50 mg of P5C and coupled it with keyhole limpet hemocyanin (KLH) to make the corresponding antigen along with BSA coupled with KLH as a control. Then we immunized mice with antigens to induce sensitized B cells. After cell fusion and screening, we injected the cells into mice’ enterocoelia to induce ascites and extracted ascites to get antibodies. The concentration of P5C antibody used in cell experiments was 60 µg/ml.

### Immunofluorescence (IF)

After SHP1 knockdown, Jurkat cells were fixed with 4% paraformaldehyde (Fisher; Fair Lawn, New Jersey) for 20 min at room temperature, and blocked with normal goat serum. Primary antibodies PKM2 (3827, CST Technology)were used at 1:200 dilutions and Cy3-conjugated anti-rabbit Abs (1:2000 dilution) were used as secondary antibodies. The subsequent counterstained nuclei was 4, 6-diamidino-2-phenylindole (DAPI).

Formalin-fixed and paraffin-embedded tissue sections (5 μm) were dewaxed with xylene and rehydrated through an ethanol gradient into water. Following blocking endogenous peroxidase activity with 0.3% hydrogen peroxide for 10 min, the sections were washed with PBS and incubated overnight with primary antibodies. Primary antibodies CD3 (sc20047, Santa Cruz Biotechnology) and SHP1 (24546-1-AP, Proteintech) were used at 1:200 dilutions and PE/FITC-conjugated anti-rabbit Abs (1:2000 dilution) were used as secondary antibodies. The subsequent counterstaining for nuclei was DAPI.

All the images were acquired with an Olympus FV3000RS fluorescence microscope system.

### Extracellular acidification rate (ECAR) measurements

ECAR assays were performed using the Seahorse XF24 analyzer (Seahorse Bioscience, Agilent) according to the manufacturer’s instructions. Briefly, 2 × 10^5^ cells/well were seeded in a 24-well XF cell culture microplate in culture medium 24 h before the assay. ECAR was measured with an XF24 analyzer in XF base medium (pH 7.4) containing 1 mM glutamine following sequential additions of glucose (10 mM), oligomycin (1 mM) and 2-DG (50 mM). Data were analyzed using the Seahorse XF Glycolysis Stress Test Report Generator package with results normalized against cell numbers determined by Countstar BioTech Automated Cell Counter.

### Oxygen consumption rate (OCR) measurements

OCR assays were performed using the Seahorse XF24 analyzer (Seahorse Bioscience, Agilent) according to the manufacturer’s instructions. Briefly, 2 × 10^5^ cells/well were seeded in a 24-well XF cell culture microplate in culture medium 24 h before the assay. OCR was measured with an XF24 analyzer in XF base medium (pH 7.4) containing 1 mM glutamine following sequential additions of oligomycin (1 mM), FCCP (0.5 mM), and Rot + AA (0.5 mM). Data were analyzed using the Seahorse XF Cell Mito Stress Test Report Generator package with results normalized against cell numbers determined by Countstar Bio Tech Automated Cell Counter.

### Animal model

All experiments were performed under the guidelines of Hubei University of Medicine animal use regulations and approved by the institutional animal care and use committee (IACUC) at Hubei University of Medicine. 6 weeks old male C57BL/6 mice were inoculated subcutaneously with 5 × 10^6^ RM-1 cells. After 10 days, 100% of the mice grew visible tumors. The mice were randomized and assigned to the control, P5C and P5C antibody groups. The tumor volumes were calculated every 3 days using the following equation: tumor volume (mm^3^) = 1/2× (tumor length) × (tumor width)^2^. The weight of the mice was also recorded every 3 days. P5C (100 nM, 50 µL) and P5C antibody (600 µg/ml, 50 µL) solution were intratumorally injected every 3 days for a total of 18 days when the tumor diameter reached 5–7 mm. At the end of the experiment, tumors were excised, measured, and then each tumor was fixed in 4% paraformaldehyde for determining T cells infiltration.

### Statistical analysis

All experiments were performed at least three separate times with data obtained from triplicate wells in each experiment. Data are expressed as means ± SD. The statistical differences between two groups were analyzed by an unpaired Student’s t-test (two-tailed); multiple groups were compared using one-way analysis of variance (GraphPad Prism 8.0; GraphPad Software; GraphPad, Bethesda, MD). A value of *P* < 0.05 was considered significant.

## Results

### SHP1 participates in energy and carbohydrate metabolism and combines with PKM2 and LDHB

**Fig. 1:**
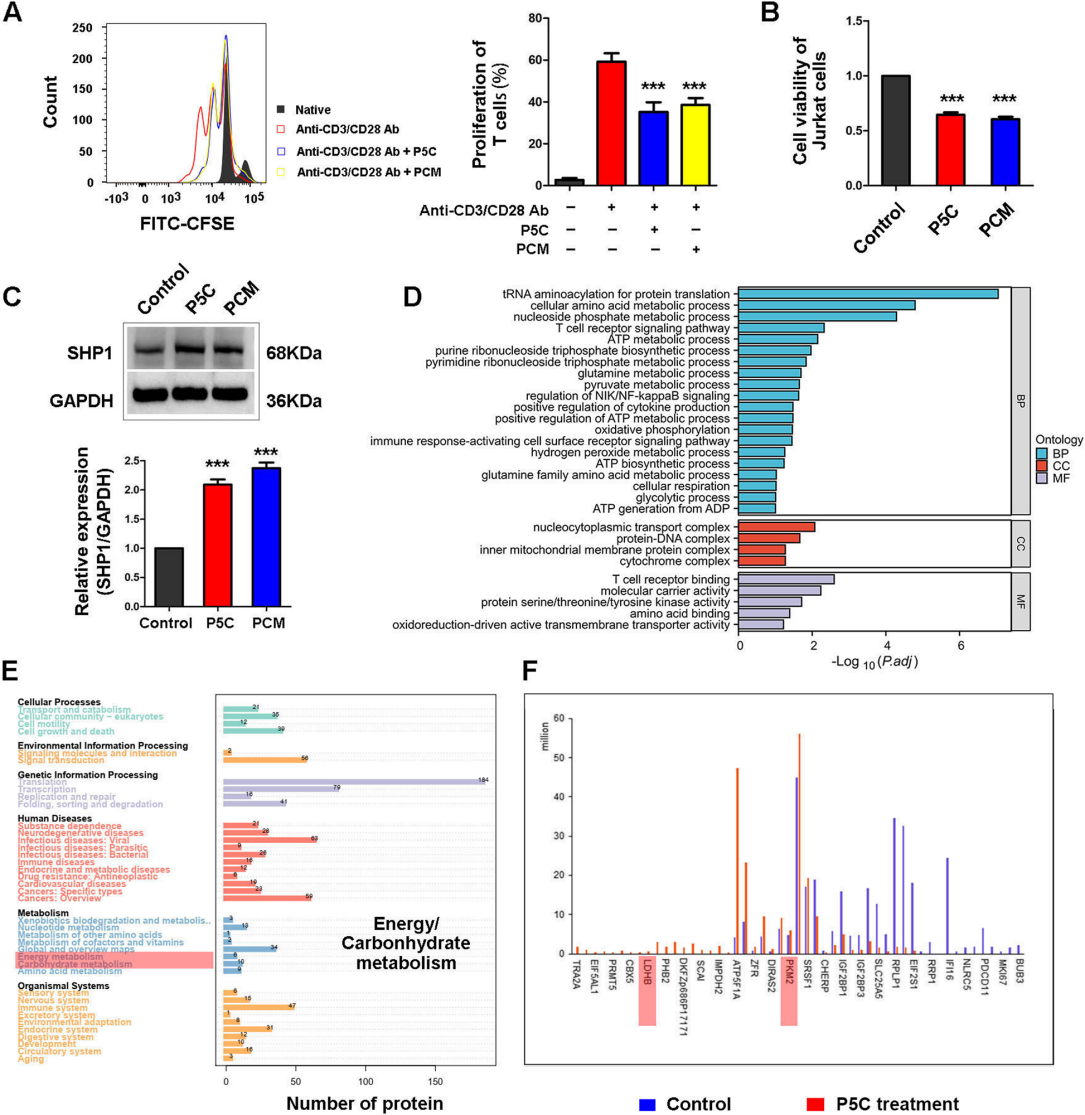
P5C inhibits T cells proliferation and SHP1 participates in energy and carbonhydrate metabolism. **A** CFSE-labeled human primary CD3^+^ T cells were pretreated with PCM or P5C for 3 days with anti-CD3/CD28 Ab. T-cell proliferation was evaluated by FACS analysis. The right side of graph is the representative result of CD3^+^ T cells proliferation. **B** Jurkat cells were treated with PCM or P5C for 72 h. Shown is the percentage of cell proliferation by CCK-8 assay. One representative experiment out of three performed. **C** Western bolt showing the protein expression of SHP1 in Jurkat cells after treatment with PCM or P5C. An antibody to GAPDH was used as a loading control. **D** SHP1 was captured from Jurkat cells after treated with P5C, and all the proteins bound with SHP1 were checked by LC/MS-MS. Through GO analysis, differential GO term in biological process (BP), cellular component (CC) and Molecular Function (MF) are shown. **E** All the proteins bound with SHP1 were summarized and shown in KEGG pathway. **F** The proteins bound with SHP1 and involved in glucose metabolism are shown. PKM2 and LDHB are marked by red rectangle. Error bars are SEM of biological replicates and ^***^p < 0.01

In our previous study, we identified prostate cancer cells medium (PCM) contains P5C, which is harmful to T cells [4]. To confirm this finding, we reused PCM containing P5C to treat CD3^+^ T cells and Jurkat cells, which significantly inhibited the proliferation of CD3^+^ T cells and Jurkat cells (Fig. 1A, B). Furthermore, the levels of cytokines produced by CD3^+^ T cells, such as IL-2, IL-4, IL-6, IL-10, TNF-α, IFN-γ and IL-17 were decreased by PCM containing P5C (Figure S1A). Meanwhile, we also detected the expression of SHP1 in Jurkat cells and verified that SHP1 is upregulated by PCM containing P5C (Fig. 1C). We previously demonstrated that SHP1 is a key molecule involved in P5C-mediated T cell inhibition. Hence, we captured SHP1 from Jurkat cells, and examined the associated proteins by LC/MS-MS. After analyzing all the proteins bound to SHP1, we examined their subcellular localization (Figure S1B). The proportion of nuclear proteins, cytoplasmic proteins and mitochondrial proteins was 54.59%, 22.37% and 7.68% respectively. Moreover, IPR annotation and KOGs function classes are shown in Figure S1C and D. For GO term analysis, we found that the proteins associated with SHP1 participate in the process of ATP metabolism and generation, as well as glycolysis (Fig. 1D). In the KEGG pathway, we recognized that proteins bound to SHP1 are involved in energy and carbohydrate metabolism (Fig. 1E). Therefore, we speculated that SHP1 should participate in glycolysis and carbohydrate metabolism of T cells. Then we summarized the proteins and enzymes that are involved in glycolysis, and we found that PKM2 and LDHB associate with SHP1, which are crucial enzymes in glycolysis (Fig. 1F).

### SHP1, PKM2 and LDHB associate with each other and P5C inhibit T cell glycolysis

**Fig. 2:**
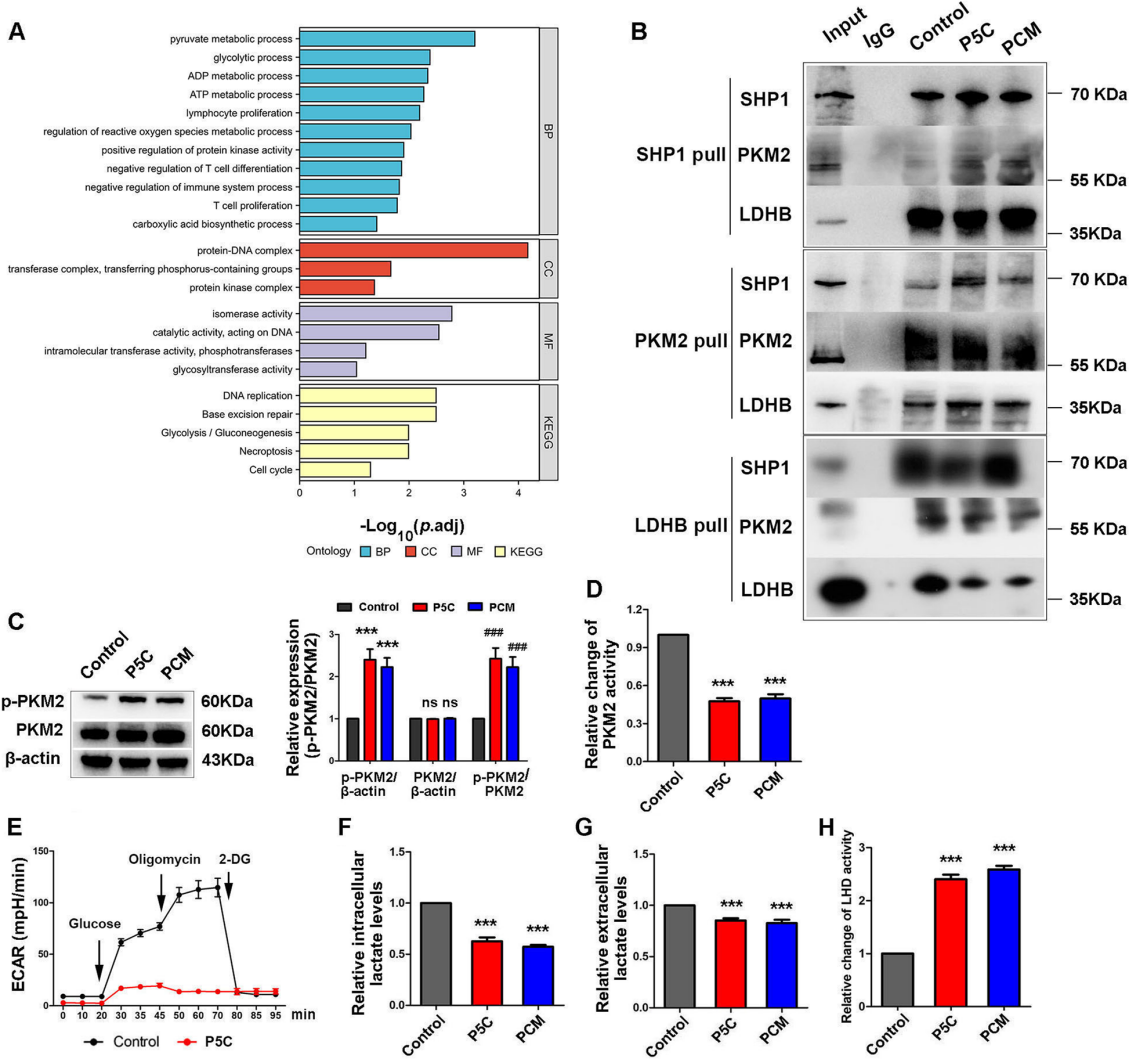
SHP1, PKM2 and LDHB bind with each other and P5C inhibits T cell glycolysis. **A** PKM2 was captured from Jurkat cells after treated with P5C, and all the proteins bound with PKM2 were checked by LC/MS-MS. All the proteins bound with PKM2 were summarized and shown in GO term and KEGG pathway. **B** SHP1, PKM2 and LDHB were pulled down by co-IP, and these three proteins bound with each other were shown by western bolt. **C** Western bolt showing the protein expression of PKM2 and p-PKM2 in Jurkat cells after treatment with PCM or P5C. An antibody to β-actin was used as a loading control. **D** Jurkat cells were treated with PCM or P5C for 24 h. Shown is the relative activity of PKM2. **E** Jurkat cells were treated with P5C, and ECAR assays were performed using the Seahorse XF24 analyzer. **F** Jurkat cells were treated with PCM or P5C for 24 h. Shown is the relative intracellular lactate levels. **G** Jurkat cells were treated with PCM or P5C for 24 h. Shown is the relative extracellular lactate levels. **H** Jurkat cells were treated with PCM or P5C for 24 h. Shown is the relative activity of LDH. Error bars are SEM of biological replicates and ^***^, ^###^*p* < 0.01

We also captured PKM2 from Jurkat cells, and checked all the associated proteins by LC/MS-MS. Using GO term analysis, we uncovered that the proteins bound to PKM2 participate in ATP metabolism and glycolysis (Fig. 2A). As PKM2 regulates T cell proliferation, it suggested that PKM2 may be involved in T cell growth by regulating glycolysis. GO term and KEGG pathway of all the proteins associated with PKM2 are shown in Figure S2A and B. We also examined the subcellular localization of all the proteins bound to PKM2 (Figure S2C). Moreover, IPR annotation and KOGs function classes are shown in Figure S2D and E. This allowed us to better understand the underlying molecular mechanisms of PKM2. We indeed uncovered that the proteins associated with PKM2 were involved in glycolysis (Fig. 2A). We subsequently captured SHP1, PKM2, and LDHB, and examined whether these three proteins associated with each other. As expected, we verified SHP1, PKM2 and LDHB could bind each other by western blotting (Fig. 2B), in accordance with our proteomics data. Therefore, we envision that SHP1-PKM2-LDHB complexes may be responsible for T cell glycolysis. We thus examined the expression of PKM and p-PKM2 in Jurkat cells treated with PCM and P5C. Our data revealed that while the expression of PKM2 does not change, the p-PKM2 level was elevated by PCM and P5C (Fig. 2C). As PKM2 is known to be inhibited by intracellular ROS via phosphorylation [18], we speculated that P5C also promotes the phosphorylation of PKM2 to inhibit its activity during glycolysis. Therefore, we investigated the activity of PKM2, which uncovered that PKM2 could be inhibited by PCM and P5C significantly (Fig. 2D).

Next, the extracellular acidification rate (ECAR) was measured to evaluate cell glycolysis. Remarkably, Jurkat cells treated with P5C showed very low ECAR, which meant cell glycolysis was inhibited infinitely (Fig. 2E). Meanwhile, we detected the content of intracellular and extracellular lactate. The levels of intracellular and extracellular lactate were both decreased by P5C (Fig. 2F, G). Then we also checked the activity of LDH, which preferentially converts lactate to pyruvate. LDH activity was promoted by P5C (Fig. 2H). This phenomenon indicated that LDH also decreased the levels of lactate. On the other hand, the expression of LDHA and LDHB in Jurkat cells was not influenced by P5C (Figure S2G, H). Relative to extremely low ECAR, interestingly the oxygen consumption rate (OCR) was not retarded, instead it was slightly accelerated (Figure S2F).

### P5C alter the metabolites in T cells detected by LC/MS-MS

**Fig. 3:**
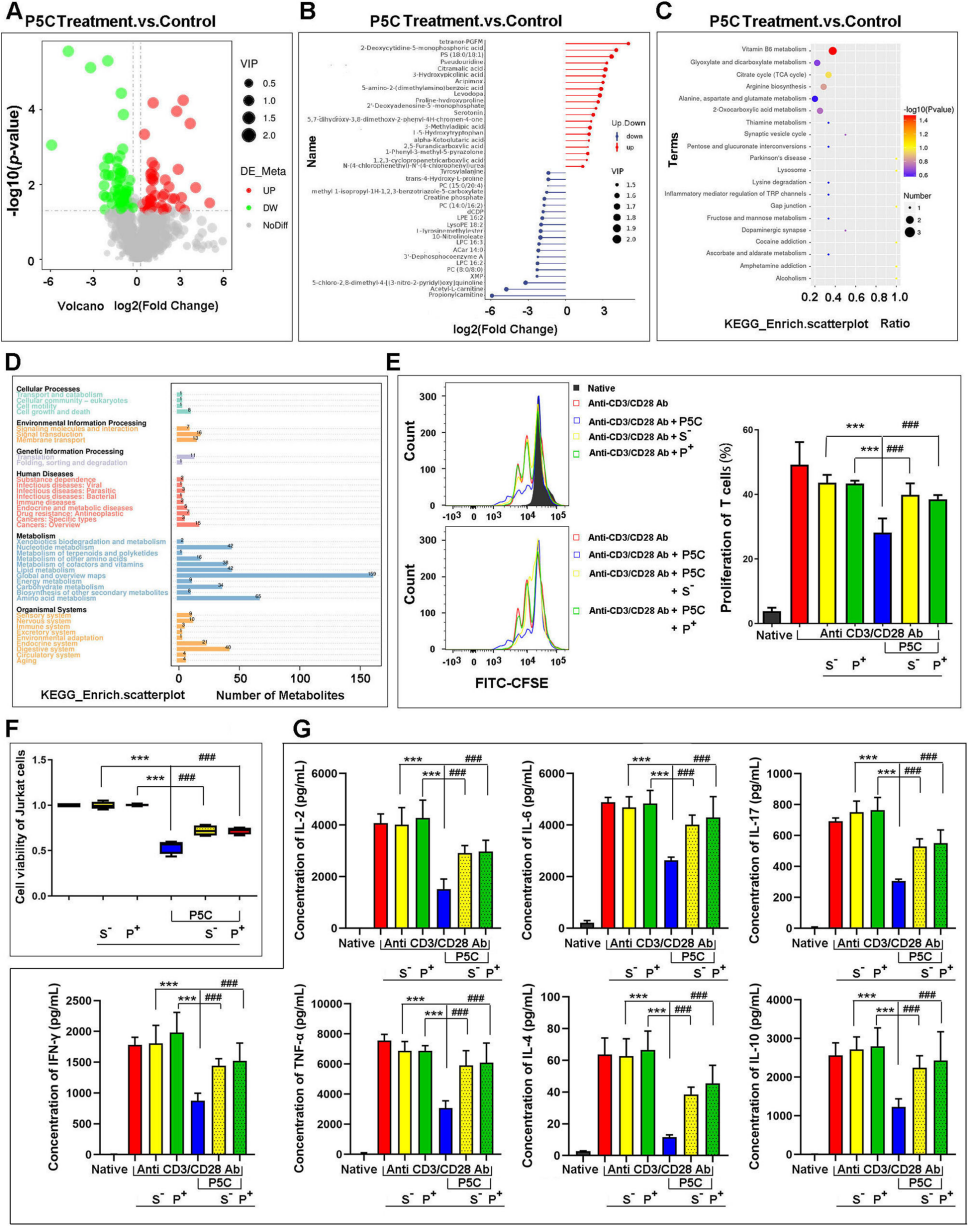
P5C alter the metabolites in T cells and PKM2 activator and SHP1 inhibitor weaken P5C effect. **A** Jurkat cells were treated with P5C and the metabolites were analyzed by LC/MS-MS. The amount and species of difference metabolites in T cells are shown as volcano map. Red dot represents increased metabolites and green dot represents decreased metabolites. **B** A list of vastly different metabolites is shown, including increased and decreased metabolites. **C** The multiple metabolic pathways that different metabolites participate in are shown as bubble diagram. **D** All the different metabolites were summarized and shown in KEGG pathway. **E** Human primary CD3 + T cells were pretreated with P5C and PKM2 activator (P^+^) piperazine or SHP1 inhibitor (S^−^) TPI-1 then stimulated for 3 days with anti-CD3/CD28 beads. Shown is the percentage of cell proliferation by flow cytometry. The left side of graph is the representative result by flow cytometry. **F** Jurkat cells were pretreated with P5C and PKM2 activator (P^+^) piperazine or SHP1 inhibitor (S^−^) TPI-1 for 3 days. Shown is the cell viability by CCK-8. **G** Human primary CD3 + T cells were pretreated with P5C and PKM2 activator (P^+^) piperazine or SHP1 inhibitor (S^−^) TPI-1 then stimulated for 3 days with anti-CD3/CD28 beads. Supernatants from cell cultures were analyzed for cytokines levels using Cytometric Beads Array, including IL-2, IL-4, IL-6, IL-10, TNF-α, IFN-γ and IL-17. Error bars are SEM of biological replicates and ^***^, ^###^*p* < 0.01

We compared all the metabolites in Jurkat cells and P5C-treated Jurkat by LC/MS-MS. We observed different metabolites included lipids (45.32%), organic acids (20.20%) and nucleosides (11.21%) were changed significantly (Figure S3B). The amount and species of different metabolites in T cells are shown as a volcano map (Fig. 3A) and heat map (Figure S3C) respectively. We show a list of vastly different metabolites in Fig. 3B, including increased and decreased metabolites. Specifically we observed that these different metabolites were involved in multiple metabolic pathways including vitamin B6 metabolism, glyoxylate and dicarboxylate metabolism, TCA cycle and arginine biosynthesis (Fig. 3C). Nevertheless, glyoxylate and dicarboxylate metabolism and TCA cycle belong to energy and carbohydrate metabolism. In the KEGG pathway, we recognized 43 metabolites participate in energy and carbohydrate metabolism, 65 metabolites in amino acid metabolism and 42 in lipid metabolism (Fig. 3D). All of these data indicate that P5C has a huge influence on T cell metabolism, especially in energy and carbohydrate metabolism.

### P5C effect could be reversed by PKM2 activator and SHP1 inhibitor

As the activity of PKM2 was reduced and expression of SHP1 was elevated, we used PKM2 activator (P^+^) piperazine (1nM) and SHP1 inhibitor (S^−^) TPI-1 (1nM) to prevent the effect of P5C on T cells. Using a dose-dependent study, we initially determined the optimal concentration, which does not affect T cells’ proliferation (Figure S3D, E). We subsequently observed that the effect of P5C could be reversed by piperazine and TPI-1, including T cell proliferation and cytokine production. After CD3^+^T cells were activated by CD3/CD28 antibodies, P^+^ and S^−^ had no effect on cell proliferation, while P5C could still suppress cell growth. Nevertheless, if we used P5C with P^+^ or S^−^ to treat CD3^+^T cells, we observed that P5C lost its ability to inhibit cell growth (Fig. 3E). Meanwhile, the same phenomenon was observed in Jurkat cells that P5C also could not suppress cell growth in the presence of P^+^ or S^−^ (Fig. 3F). Next, we also checked cytokine production in CD3^+^ T cells, including IL-2, IL-4, IL-6, IL-10, TNF-α, IFN-γ and IL-17. Similarly, P^+^ and S^−^ had no effect on cytokine production, while P5C could significantly decrease the levels of these 6 cytokines. However, P5C also lost the ability to subdue the cytokine production of CD3^+^ T cells in the presence of P^+^ or S^−^ (Fig. 3G).

### SHP1 knockdown dampens P5C effect on T cells

**Fig. 4:**
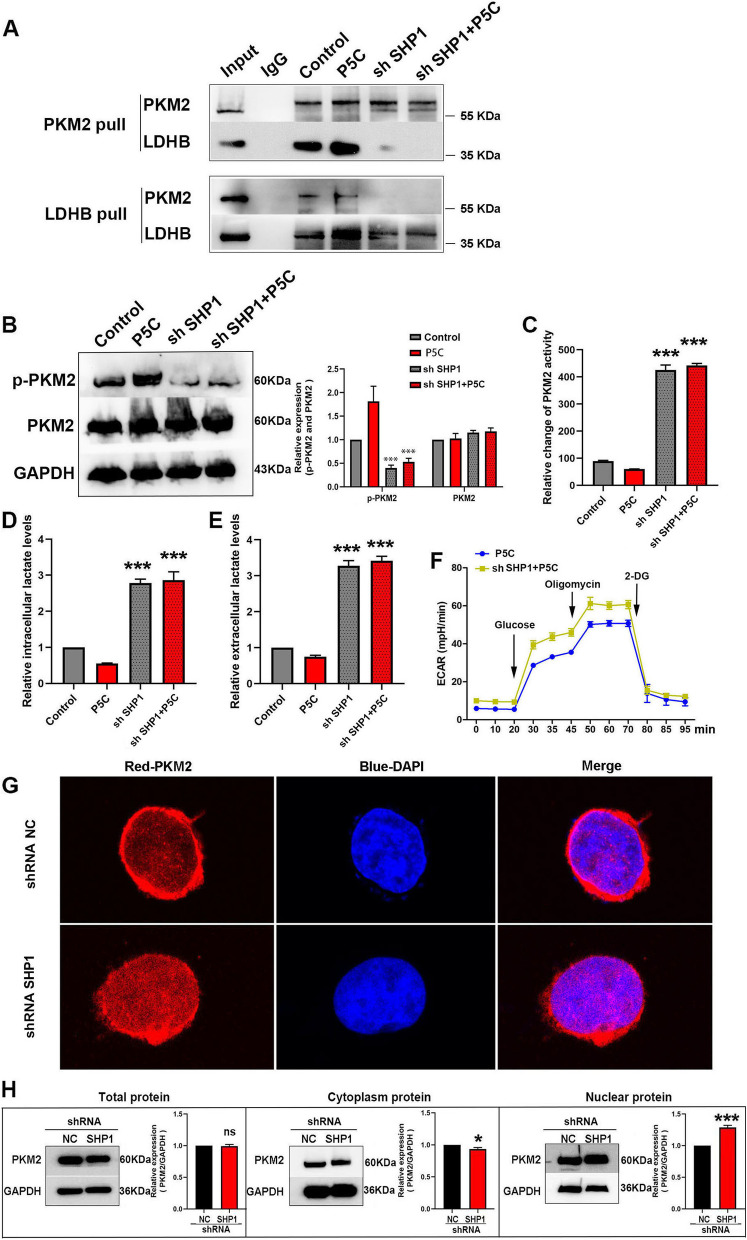
SHP1 knockdown dampens P5C effect on T cells. **A** After SHP1 knockdown, PKM2 and LDHB were captured from Jurkat cells treated with P5C by co-IP. Western bolt showing whether PKM2 and LDHB bind with each other in the absence of SHP1. **B** After SHP1 knockdown, western bolt showing the protein expression of PKM2 and p-PKM2 in Jurkat cells treated with P5C. An antibody to GAPDH was used as a loading control. **C** After SHP1 knockdown, Jurkat cells were treated with P5C for 24 h. Shown is the relative activity of PKM2. **D** After SHP1 knockdown, Jurkat cells were treated with P5C for 24 h. Shown is the relative intracellular lactate levels. **E** After SHP1 knockdown, Jurkat cells were treated with P5C for 24 h. Shown is the relative extracellular lactate levels. **F** After SHP1 knockdown, Jurkat cells were treated with P5C, and ECAR assays were performed using the Seahorse XF24 analyzer. **G** After SHP1 knockdown, IF showing the location of PKM in Jurkate cells. **H** Western blot showing the total, cytoplasmic and nuclear protein expression of PKM2 in Jurkat cells upon SHP1 knockdown. Error bars are SEM of biological replicates and ^***^p < 0.01

In order to ascertain whether SHP-PKM2-LDHB complexes play a crucial role in the process of P5C-mediated inhibition of T cells’ glycolysis, we knocked down the expression of SHP1 in Jurkat cells. Firstly, we designed three shRNAs and verified shRNA-1 most efficiently knocked down SHP1 (Figure S4B). After knockdown, we captured PKM2 and LDHB from Jurkat cells, and examined the association of PKM2 and LDHB. Astonishingly, we found that PKM2 and LDHB did not bind to each other (Fig. 4A), which suggested that SHP1 could be the bridge between PKM2 and LDHB. Simultaneously, the expression of PKM2 and p-PKM2 was also detected by western blotting. As expected, the expression of PKM2 did not change when cells were exposed to P5C and SHP1 shRNAs (Fig. 4B). Nevertheless, the levels of p-PKM2 showed an enormous reduction upon SHP1 knock-down (Fig. 4B). Otherwise, P5C promoted p-PKM2 levels, but lost the ability upon SHP1 knockdown (Fig. 4B). Furthermore, we also examined the activity of PKM2 as the enzyme in glycolysis. SHP1 depletion remarkably elevated the activity of PKM2, because P5C lost the capacity to inhibit the activity of PKM2 upon SHP1 knockdown (Fig. 4C). The reduction in p-PKM2 levels signified that the activity of PKM2 was enhanced. Therefore, our results indicated that SHP1 owns the capacity to restrain PKM2, and SHP1 knockdown causes the incapacitation of P5C on PKM2 activity. Subsequently, the levels of intracellular and extracellular lactate were driven by PKM2 activity. SHP1 depletion solely increased the levels of lactate, while P5C decreased them and SHP1 knockdown weakened the ability of P5C (Fig. 4D, E). We verified P5C could inhibit glycolysis. We also observed that SHP1 shRNA promoted glycolysis, as shown by ECAR (Fig. 4F). SHP1 shRNA reduced OCR, whereas P5C promoted it (Figure S4C). As SHP1 suppresses glycolytic PKM2 activity, we inspected the location of PKM2 after knocking down SHP1, and we found that PKM2 intranuclear localization was enhanced (Fig. 4G), which indicated that SHP1 not only adjusts glycolytic PKM2 activity but also regulates PKM2 location. PKM2 in the dimer state can enter the nuclear to regulate gene expression and glycolysis [19]. We also detected the expression by western blot, which was consistent with IF, which showed the expression of PKM2 in nucleus increased and in cytoplasm decreased (Fig. 4H). This showed that PKM2 could translocate into the nucleus to regulate gene expression and glycolysis in the absence of SHP1.

To explore the relationship of SHP1, PKM2 and LDHB, we speculated that SHP1 may be involved in the glucose metabolism of T cells. We compared all the metabolites in Jurkat cells and SHP1 knocked down Jurkat cells by LC/MS-MS. The amount and species of difference metabolites in T cells are shown as a volcano map (Figure S5B) and heat map (Figure S5C) respectively. In the volcano map, we showed 577 discrepant metabolites, including 228 increased and 349 decreased metabolites. In the heat map, we showed discrepant metabolites in different species. The red rectangle highlighted the carbohydrates and its metabolites. There were 55 discrepant carbohydrates and its metabolites after SHP1 knockdown. We listed all the 55 metabolites in Figure S5D. Through metabolomics analysis, we found that the metabolism of T cells changed in the absence of SHP1, including glucose metabolism.

### P5C antibody restores T cells proliferation and function in vitro

**Fig. 5:**
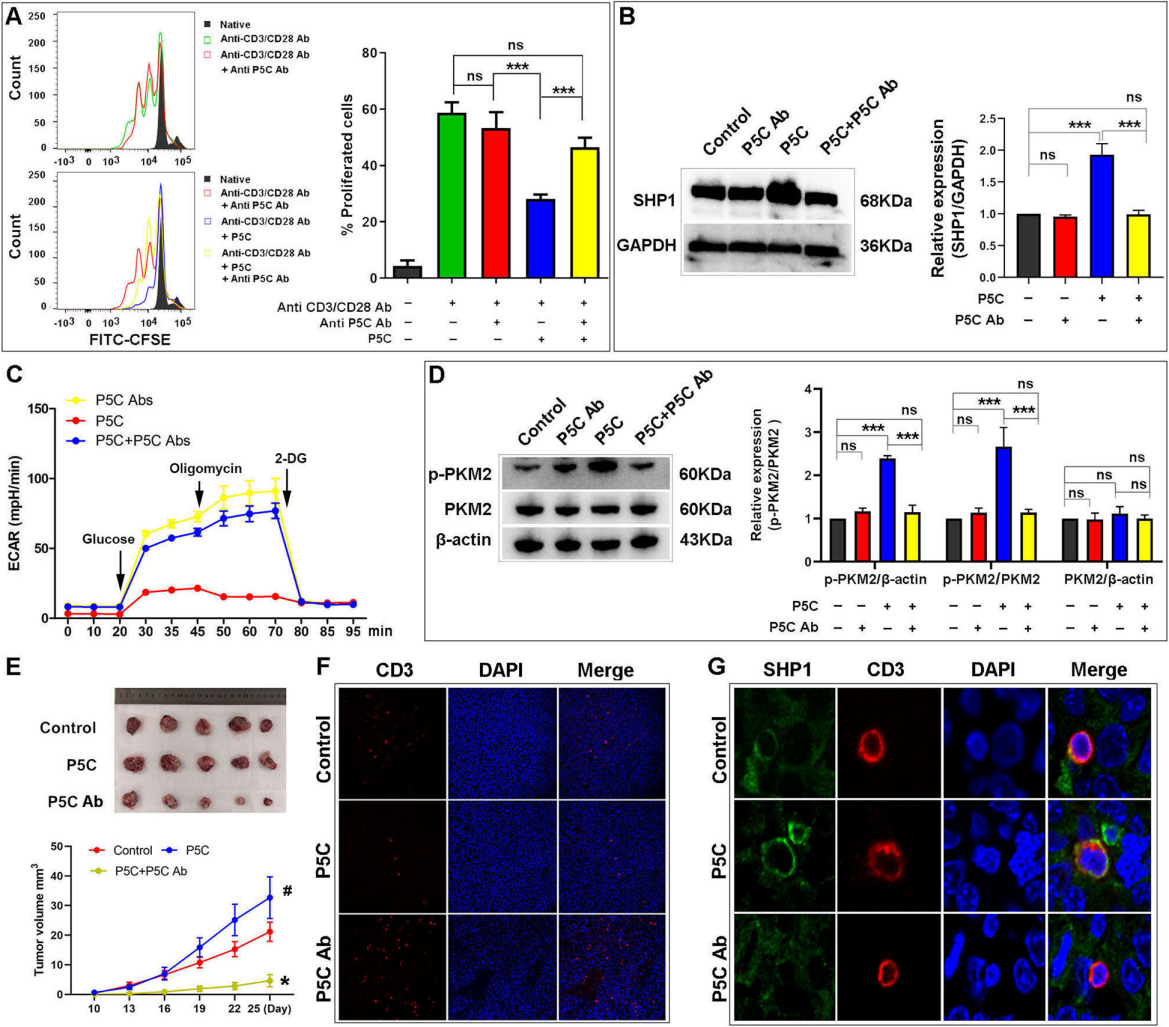
P5C antibody counter the effect of P5C in vitro and in vivo. **A** Human primary CD3^+^ T cells were pretreated with P5C and P5C Ab then stimulated for 3 days with anti-CD3/CD28 beads. Shown is the percentage of cell proliferation by flow cytometry. The left side of graph is the representative result by flow cytometry. **B** Jurkat cells were pretreated with P5C and P5C Ab for 24 h. Western bolt showing the protein expression of SHP1 in Jurkat cells. An antibody to GAPDH was used as a loading control. **C** Jurkat cells were treated with P5C and P5C Ab, and ECAR assays were performed using the Seahorse XF24 analyzer. **D** Jurkat cells were pretreated with P5C and P5C Ab for 24 h. Western bolt showing the protein expression of PKM2 and p-PKM2 in Jurkat cells. An antibody to β-actin was used as a loading control. **E** Mice were inoculated subcutaneously with 1 × 10^6^ RM-1 cells to construct animal model, and treated with P5C and P5C Ab. The picture shows the tumors after harvesting. The line graph below shows the mean of tumor volume measured at the indicated number of days after mice were treated. **F** The infiltration of CD3^+^ T cells in tumor tissue detected by IF. The red light marked T cells. **G** IF showing SHP expression in CD3^+^ T cells. DAPI was used for marking nuclear. The red light marked T cells and green light marked SHP1. Error bars are SEM of biological replicates and ^*^, ^#^*p* < 0.05; ^***^p < 0.01

In order to intercept P5C, we produced an antibody for targeting P5C by monoclonal technique. We subsequently examined the proliferation of CD3^+^ T cells (Fig. 5A) and Jurkat cells (Figure S6A). We observed that P5C antibody (P5C Ab) could inhibit the effect of P5C on cell proliferation significantly. Similarly, P5C Ab also reversed the effect of P5C on cytokine production (Figure S6B). We also evaluated SHP1 expression, and we found that P5C Ab recovered it which was elevated by P5C (Fig. 5B). To elucidate cell glycolysis, ECAR was also examined. We showed that P5C Ab accelerated ECAR to counter the effect of P5C (Fig. 5C). Accordingly, P5C Ab also led to the recovery of glycolysis. We also checked the expression of PKM2/p-PKM2 and the activity of PKM. The expression of PKM2 did not change as always, but P5C Ab restored the expression of p-PKM2 (Fig. 5D) and activity of PKM2 (Figure S6C). Likewise, the variation trend of the levels of intracellular and extracellular lactate was in accord with the activity of PKM2. Thus, P5C Ab increased the content of lactate, which was decreased by P5C (Figure S6D, E).

### P5C antibody inhibits prostate tumor growth and increases T cells infiltration on animal model

Because of the defectiveness of T cells in nude mice, we could not use human prostate cancer cells to conduct a xenograft model to verify P5C Ab in vivo. We chose murine prostate cancer cells RM-1 to construct an animal model. Strikingly, P5C and P5C Ab influenced tumor growth in an animal model. P5C promoted tumor growth and P5C Ab reversed it (Fig. 5E). Furthermore, P5C decreased T cells infiltration and P5C Ab accelerated T cells infiltration (Fig. 5F). This phenomenon illustrated that P5C Ab created a favorable environment for T cells in the tumor environment. Next, we also observed SHP1 expression in T cells by IF, and found that P5C Ab recovered it, which was elevated by P5C (Fig. 5G).

We verified the availability of P5C Ab in vitro and in vivo, and found that P5C Ab could counter P5C to recover T cells proliferation and cytokine secretion, and also accelerate T cells infiltration in the tumor environment. This finding provided us with a prospect for using P5C Ab for prostate cancer treatment.

## Discussion

The metabolism of tumor cells differs from healthy or normal mammalian cells due to the acquisition and maintenance of malignant properties [20, 21]. Tumor cells undergo metabolic reprogramming to fulfill the energy needs for tumor growth and progression in the tumor microenvironment (TME) [22]. The metabolic stress occurring in TME hampers T cell anti-tumor immunity by disturbing T cell metabolic and epigenetic programs [23]. Hypoxia is one of the most prominent features of most solid tumors, which results from the uncontrolled proliferation of tumor cells, resulting in low oxygen supply in the TME [23]. Hypoxia could reduce succinate production, which not only limits the infiltration of CD8^+^ T cells into the TME but also drives terminal differentiation of CD8^+^ T cells [24–27]. To provide the anti-tumor effect, T cells engage in aerobic glycolysis to maintain a higher proliferation rate and increased cytokine secretion. However, the level of glucose within the TME is usually low in general solid tumors. Furthermore, metabolic byproducts such as lactate are accumulated in the TME [28, 29]. Lactate is enriched in the TME, forms an acidic environment which consequently suppresses the proliferation and weakens the anti-tumor function of T cells by blocking the export of lactic acid from T cells and decreasing ATP production [25, 30]. During tumor growth, cancer cells increase the expression of amino acid transporters to fulfill the energy demand, thereby exhausting amino acids in the TME and inhibiting proliferation, differentiation, and functions of T cells [31, 32]. For example, a lack of alanine has been demonstrated to reduce cytokine production in CD8^+^ T cells. Similarly, tryptophan also influences the metabolism of T cells [33]. Besides limited nutrient availability, TME is also enriched in lipids, which have been reported to be harmful to T cells as well [34]. Hence, the metabolism of tumor cells has multiple detrimental effects on T cells in TME.

Previously we demonstrated that P5C released into the tumor environment by prostate cancer cells inhibits the proliferation and function of T cells by up-regulating SHP1 expression in T cells [4]. Meanwhile, P5C increases ROS production but decreases ATP production in T cells. P5C, as an intermediate in proline, glutamate, and ornithine interconversions, links the TCA cycle, urea cycle, and proline metabolism [35]. The conversion of P5C to proline is catalyzed by P5C reductase (PYCR), while proline to P5C is catalyzed by proline dehydrogenase/oxidase (PRODH/POX) [36]. P5C participates not only in the regulation of cell survival, autophagy, growth, and proliferation via involvement in the synthesis of biomass components, but also in the regulation of cell death by apoptosis, because the regulation of ROS levels relies on the activity of PRODH/POX [35]. In this work, we explored the effect of P5C on T cell metabolism in the prostate cancer cell microenvironment.

SHP1 is a protein tyrosine phosphatase expressed ubiquitously in hematopoietic cells and has been regarded as a negative regulator of T cell activation [37]. We previously affirmed that P5C could elevate the expression of SHP1 in T cells [4]. Therefore, we examined the interactome of SHP1 in T cells by proteomics analysis. We found that SHP1, PKM2 and LDHB could bind to each other. PKM2 acts as a key enzyme in glucose metabolism and can be used as a switch for energy metabolism and material synthesis [38]. Moreover, the bidirectional conversion of pyruvate and lactate could be catalyzed by lactate dehydrogenase A (LDHA) and lactate dehydrogenase B (LDHB). LDHA has a higher affinity for pyruvate, preferentially converting pyruvate to lactate, and NADH to NAD^+^ in anaerobic conditions, whereas LDHB possesses a higher affinity for lactate, preferentially converting lactate to pyruvate, and NAD^+^ to NADH, when oxygen is abundant [39]. Hence, as PKM2 and LDHB are involved in glucose metabolism, we concluded that P5C could influence the glycolysis of T cells. Our proteomics analysis indicated that energy metabolism and carbohydrate metabolism are involved in this process. By ECAR measurement on Seahorse XF96 analyzer, we verified that P5C could inhibit T cell glycolysis remarkably.

In the process of T cell activation, T cells undergo metabolic reprogramming to upregulate glycolysis, which is required to support their growth, differentiation and function [40, 41]. It is widely agreed that proliferating cells have a high rate of glycolysis. Alterations in the glycolysis of CD8^+^ T cells have an important effect on their activation and function, while glycolysis is important for CD8^+^ T cell functional failure and recovery [42]. It was reported that PD-1 ligation impedes T cell development by altering the metabolic reprogramming of activated T cells by inhibiting glycolysis [43]. Therefore, recovering glycolysis of T cells has been identified as a potential strategy for tumor immunotherapy. In our study, we considered SHP1-PKM2-LDHB complex should be responsible for the inhibition of P5C on T cell glycolysis. SHP1 knockdown triggered the separation of PKM2 and LDHB, which resulted in the recovery of T cell glycolysis causing increased proliferation and cytokine production. Otherwise, SHP1 knockdown also promoted glycolytic PKM2 activity and caused PKM2 to translocate into the nucleus. This uncovered SHP1 as a negative regulator of T cell activation, which participates in T cell glycolysis by regulating PKM2. We also used PKM2 agonist and SHP1 inhibitor, which showed that T cell glycolysis could be restored as well. Meanwhile, we also generated P5C antibody to hinder P5C from the source. Many fields have benefited from the use of antibodies to target metabolites [44–46]. We uncovered that P5C antibody could recover T cell glycolysis and facilitate proliferation and cytokine production of T cells. In a prostate cancer animal model, P5C antibody also inhibited tumor growth and increased T cells infiltration.

In conclusion, P5C released by prostate cancer cells inhibits the glycolysis of T cells in the tumor microenvironment via SHP1, PKM2 and LDHB complexes. The activity of PKM2 could be inhibited by P5C, while the activity of LDHB could be promoted, which reduced the content of intracellular lactate. Additionally, SHP1 knockdown and P5C antibody could recover T cell glycolysis and promote proliferation and function of T cells, which provides a new standpoint for tumor immunotherapy. Therefore, targeting P5C and its related pathways could be a potential therapeutic strategy for prostate cancer.

### Supplementary Information

**Additional file 1: Table S1.** SHP1 shRNA sequence. **Figure S1.** P5C inhibits T cells cytokine production and SHP1 bind proteins proteomics analysis, related to Fig. 1. **Figure S2.** PKM2 bind proteins proteomics analysis and P5C inhibits T cell glycolysis, related to Fig. 2. **Figure S3.** P5C alter the metabolites in T cells, related to Fig. 3. **Figure S4.** SHP1 knockdown dampens P5C effect on T cells, related to Fig. 4. **Figure S5.** SHP1 knockdown alter the metabolites in T cells. **Figure S6.** P5C antibody counter the effect of P5C, related to Fig. 5.

## Data Availability

No datasets were generated or analysed during the current study.
