# Supplementary material for: 1-Pyrroline-5-carboxylate inhibit T cell glycolysis in prostate cancer microenvironment by SHP1/PKM2/LDHB axis
Source: Cell Commun Signal. 2024 Feb 8;22:101. doi: 10.1186/s12964-024-01493-1 (PMC10851605; doi:10.1186/s12964-024-01493-1)
Supplement: Supplementary file 1 — Additional file 1: Table S1. SHP1 shRNA sequence. Figure S1. P5C inhibits T cells cytokine production and SHP1 bind proteins proteomics analysis, related to Fig. 1. Figure S2. PKM2 bind proteins proteomics analysis and P5C inhibits T cell glycolysis, related to Fig. 2. Figure S3. P5C alter the metabolites in T cells, related to Fig. 3. Figure S4. SHP1 knockdown dampens P5C effect on T cells, related to Fig. 4. Figure S5. SHP1 knockdown alter the metabolites in T cells. Figure S6. P5C antibody counter the effect of P5C, related to Fig. 5. [file 12964_2024_1493_MOESM1_ESM.docx]

**Supplementary Table**

Table 1 SHP1 shRNA sequence

| No. Gene TargetSeq |
| --- |
| 1. PTPN6 GGAGCATGACACAACCGAATA 2. PTPN6 GGGATCAGGTGACCCATATTC 3. PTPN6 ACTATGCCACGAGGGTGAATG   NC NC CCTAAGGTAAGTCGCCCTCG |

**Supplementary Figures**


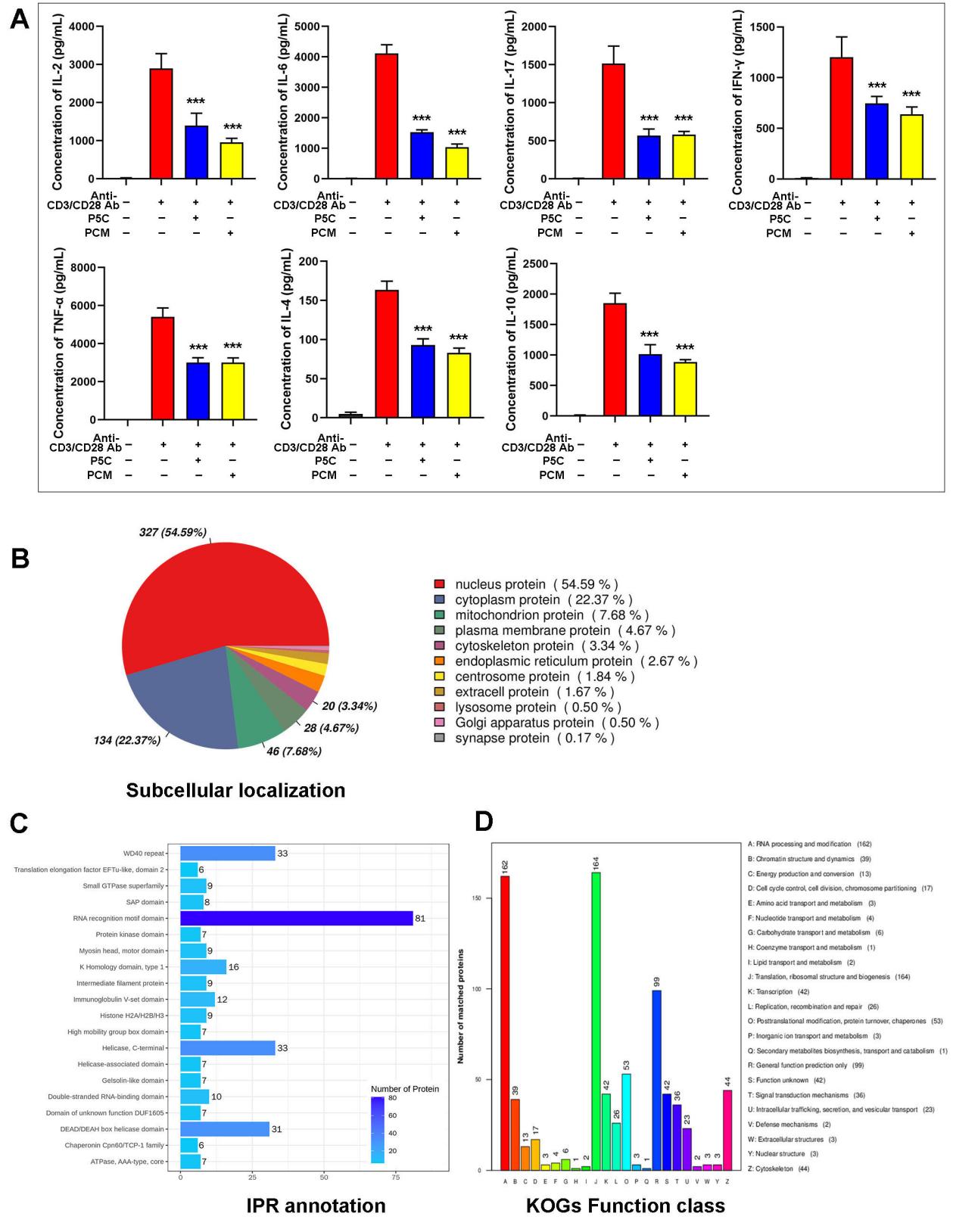


Figure S1 P5C inhibits T cells cytokine production and SHP1 bind proteins proteomics analysis, related to Figure 1

(A) Human primary CD3+ T cells were pretreated with PCM or P5C for 3 days with anti-CD3/CD28 Ab. Supernatants from cell cultures were analyzed for cytokines levels using Cytometric Beads Array, including IL-2，IL-4，IL-6，IL-10，TNF-α, IFN-γ and IL-17. (B) SHP1 was captured from Jurkat cells after treated with P5C, and all the proteins bound with SHP1 were checked by LC/MS-MS. The pie chart showing the percentage of subcellular localization of all the proteins bound with SHP1. (C) The bar graph showing the IPR annotation. (D) The bar graph showing the KOGs function class. Error bars are SEM of biological replicates and ^***^*p* < 0.01


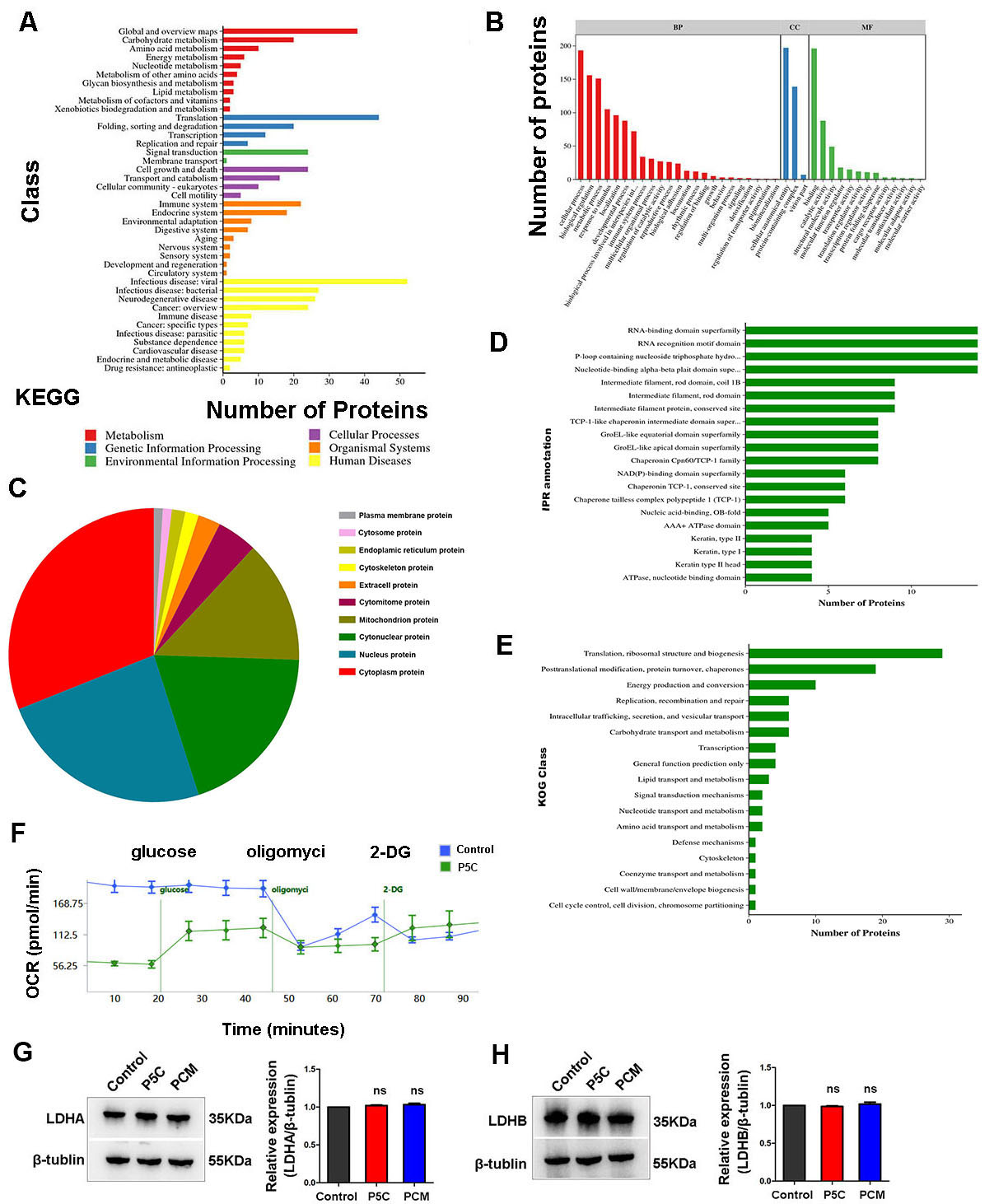


Figure S2 PKM2 bind proteins proteomics analysis and P5C inhibits T cell glycolysis, related to Figure 2

(A) PKM2 was captured from Jurkat cells after treated with P5C, and all the proteins bound with PKM2 were checked by LC/MS-MS. The bar graph showing the KEGG pathway. (B) The bar graph showing the GO term analysis. (C) The pie chart showing the subcellular localization of all the proteins bound with PKM2. (D) The bar graph showing the IPR annotation. (E) The bar graph showing the KOGs function class. (F) Jurkat cells were treated with P5C, and OCR assays were performed using the Seahorse XF24 analyzer. (G) Western bolt showing the protein expression of LDHA in Jurkat cells after treatment with PCM or P5C. An antibody to β-tublin was used as a loading control. (H) Western bolt showing the protein expression of LDHB in Jurkat cells after treatment with PCM or P5C. An antibody to β-tublin was used as a loading control.


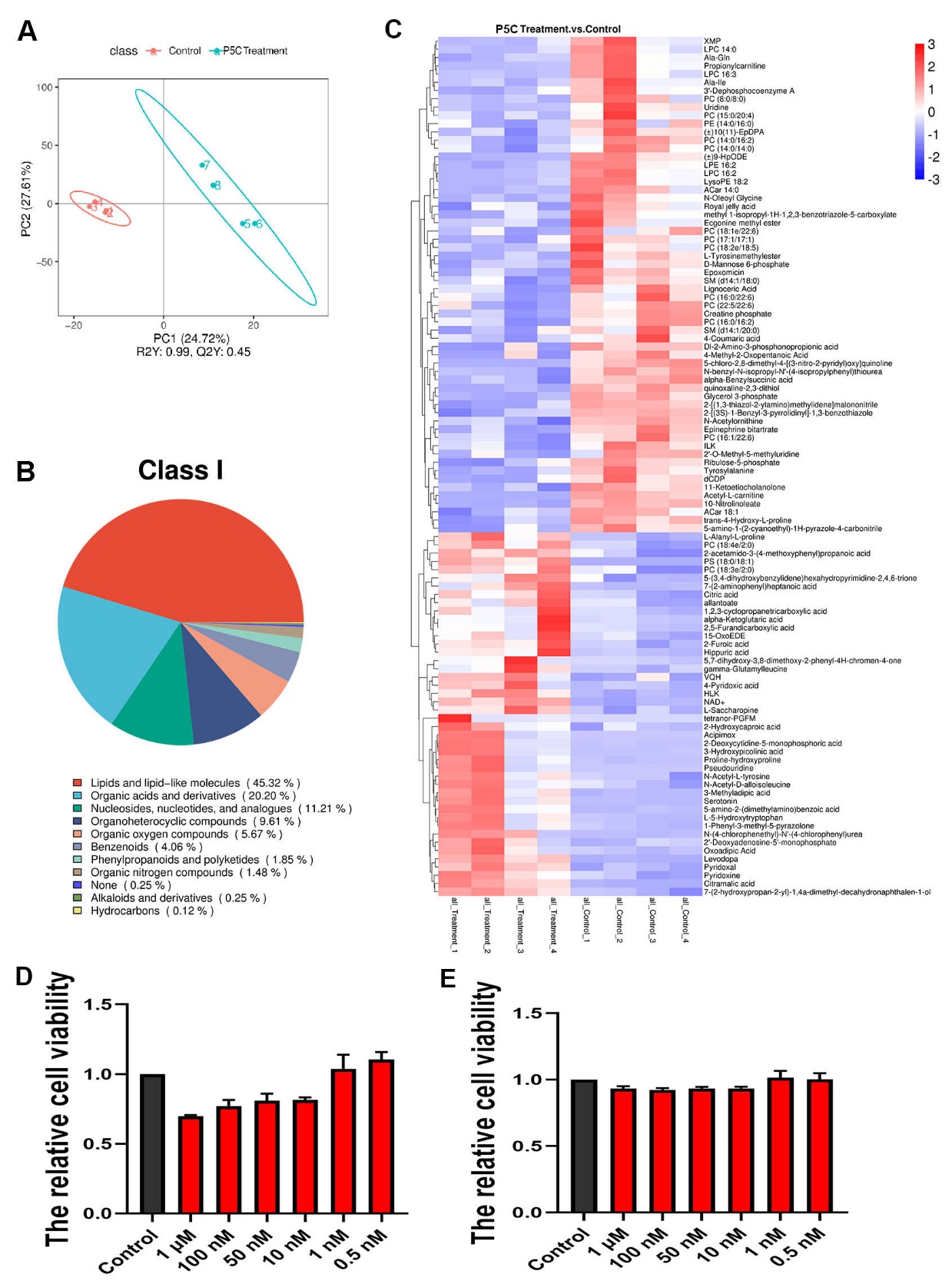


Figure S3 P5C alter the metabolites in T cells, related to Figure 3

(A) Jurkat cells were treated with P5C and the metabolites were analyzed by LC/MS-MS. The graph showing the PCA score plots. (B) The pie chart showing the percentage of species of different metabolites. (C) The heat map showing the different metabolites. (D) Jurkat cells were pretreated with SHP1 inhibitor TPI-1 for 24 h to choose the optimal concentration. Shown is the percentage of cell proliferation by CCK-8 assay. One representative experiment out of three performed. (E) Jurkat cells were pretreated with PKM2 activator piperazine for 24 h to choose the optimal concentration. Shown is the percentage of cell proliferation by CCK-8 assay. One representative experiment out of three performed.


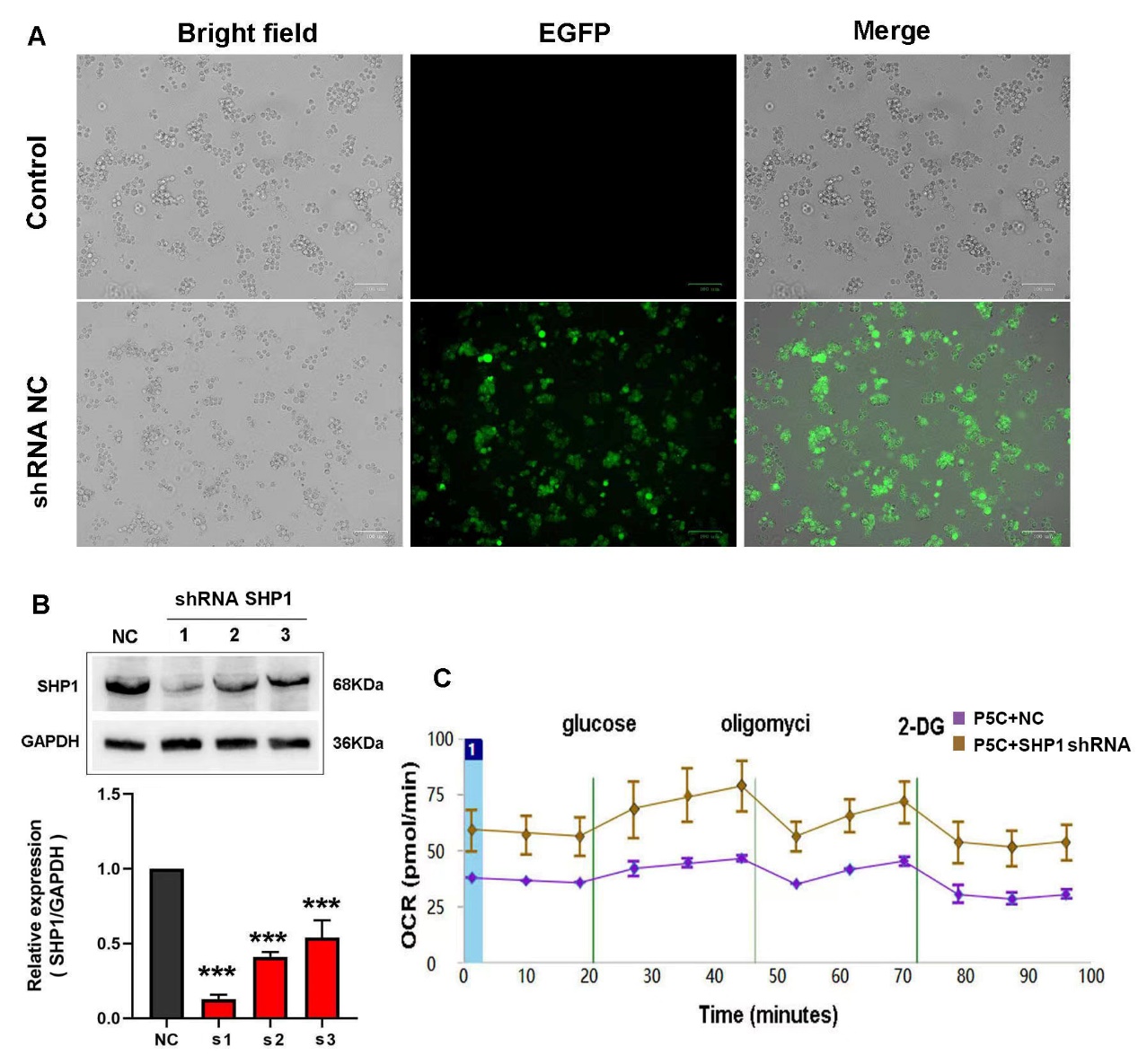


Figure S4 SHP1 knockdown dampens P5C effect on T cells, related to Figure 4

(A) After SHP1 shRNA tranfection, took the pictures by microscope and fluorescence microscope to show the transfection efficiency. (B) Western blot showing the expression of SHP1 after transfection by 3 shRNA. (C) After SHP1 knockdown, Jurkat cells were treated with P5C, and OCR assays were performed using the Seahorse XF24 analyzer. Error bars are SEM of biological replicates and ^*^*p*<0.05; ^***^*p* < 0.01


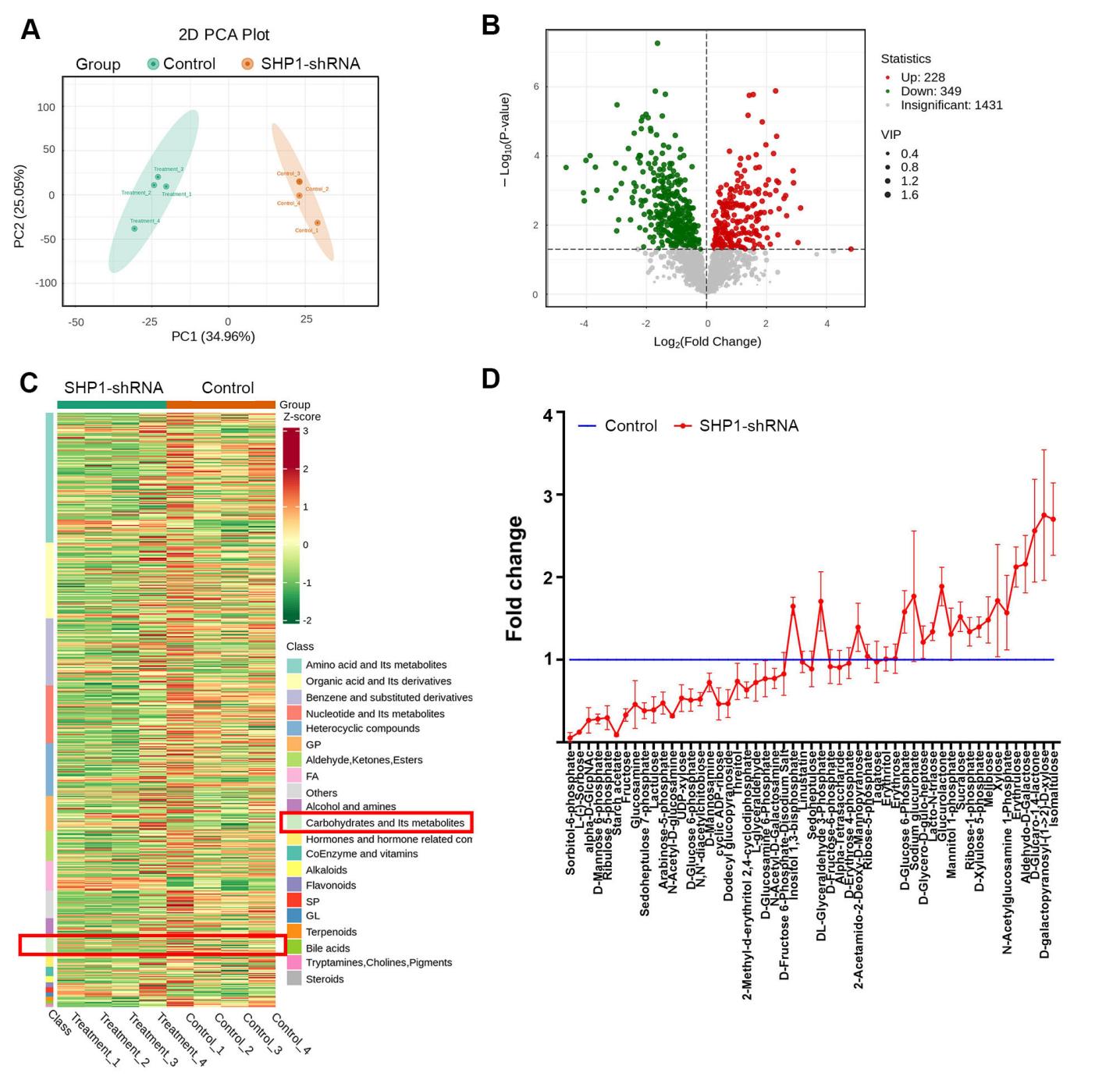


Figure S5 SHP1 knockdown alter the metabolites in T cells

(A) Jurkat cells were treated with SHP1 shRNA and the metabolites were analyzed by LC/MS-MS. The graph showing the PCA score plots. (B) The amount and species of difference metabolites in T cells are shown as volcano map. Red dot represents increased metabolites and green dot represents decreased metabolites. (C) The heat map showing discrepant metabolites in different species. The red rectangle highlights carbohydrates and its metabolites. (D) A list of 55 discrepant carbohydrates and its metabolites is shown.


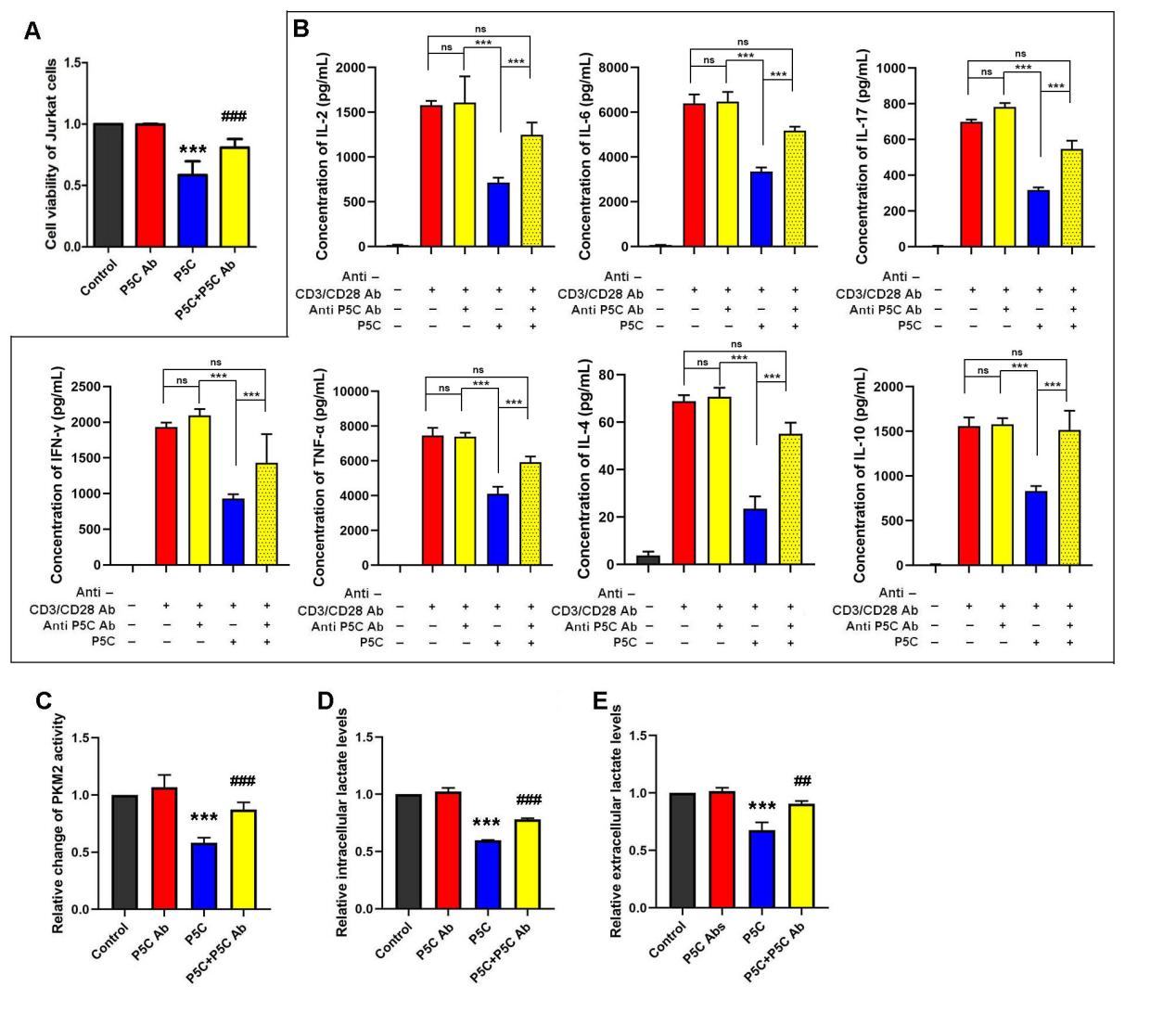


Figure S6 P5C antibody counter the effect of P5C, related to Figure 5

(A) Jurkat cells were pretreated with P5C and P5C Ab for 72 h. Shown is the percentage of cell proliferation by CCK-8 assay. One representative experiment out of three performed. (B) Human primary CD3+ T cells were pretreated with P5C and P5C Ab then stimulated for 3 days with anti-CD3/CD28 beads. Supernatants from cell cultures were analyzed for cytokines levels using Cytometric Beads Array, including IL-2，IL-4，IL-6，IL-10，TNF-α, IFN-γ and IL-17. (C) Jurkat cells were pretreated with P5C and P5C Ab for 24 h. Shown is the relative activity of PKM2. (D) Jurkat cells were pretreated with P5C and P5C Ab for 24 h. Shown is the relative intracellular lactate levels. (E) Jurkat cells were pretreated with P5C and P5C Ab for 24 h. Shown is the relative extracellular lactate levels. Error bars are SEM of biological replicates and ^***^, ^###^*p* < 0.01
